# Effect of metformin on outcome in patients undergoing primary percutaneous coronary intervention for st-segment elevation myocardial infarction

**DOI:** 10.1186/2197-425X-3-S1-A802

**Published:** 2015-10-01

**Authors:** RA Posma, E Lipsic, P van der Harst, I van der Horst

**Affiliations:** University Medical Center Groningen, Department of Critical Care, Groningen, Netherlands; University Medical Center Groningen, Department of Cardiology, Groningen, Netherlands

## Introduction

The oral antihyperglycemic agent metformin was associated with favorable outcome and smaller myocardial infarct size in patients with diabetes undergoing percutaneous coronary interventions (PCI) for ST-segment elevation myocardial infarction (STEMI) [1, 2]. However, these findings have not been validated.

## Objectives

To determine the effect of chronic metformin treatment on cardiovascular morbidity and mortality in patients with diabetes presenting with STEMI subsequently undergoing PCI.

## Methods

From January 2004 until June 2013, all consecutive critically ill patients undergoing primary PCI for STEMI at the University Medical Center Groningen were included in a registry and 1-year follow-up was obtained. Our primary endpoint consisted of the composite endpoint of myocardial infarction, target vessel and target lesion revascularization, and all-cause mortality (MACE). The secondary endpoint, myocardial infarction size, was estimated using peak levels of creatine kinase (CK), the myocardial band of CK (CK-MB), troponin T, and high-sensitive troponin T (hs-troponin T). The effect of metformin on myocardial infarct size from the 2004-2010 cohort has been reported previously [1]. Therefore myocardial infarction size was reported for patients admitted from 2011 until 2013 and the combined 2004-2013 cohort.

## Results

In total, 4776 consecutive patients underwent primary PCI for STEMI, 719 (15%) diabetic patients were included in the final analysis and 215 (30%) patients used metformin at admission. MACE and mortality rates were 21% and 12% for patients with diabetes, 23% and 19% for metformin patients, 21% and 15% for patients on sulfonylurea, and 30% and 20% for patients on insulin, respectively. Metformin was not associated with reduced risk for MACE (adjusted hazard ratio (aHR): 1.19 (95% confidence interval (95%CI) 0.78-1.81), P = 0.42) or survival benefit (aHR: 0.23 (CI95% 0.80-2.51), P = 0.23) compared to diabetic patients not using metformin. Insulin use was an independent predictor for MACE (aHR 1.73 (CI95% 1.13-2.65), P = 0.01) and all-cause mortality (aHR 1.81 (CI95% 1.03-3.21), P = 0.04). Baseline levels of CK, CK-MB, and hs-troponin T were comparable between both groups. Median (interquartile range) peak levels of CK, CK-MB, and hs-Troponin T were all non-significant lower in the metformin group (table 1). When both cohorts were combined, peak levels of CK, CK-MB, and troponin T were all significantly lower in patients using metformin, as depicted in table 1.

**Table 1:** Table 1

	2011-2013 cohort			Combined 2004-2013 cohort		
Peak value	Metformin (n = 83)	No metformin (n = 72)	P-value	Metformin (n = 254)	No metformin (n = 537)	P-value
CK (U/L)	846 (297-2317)	1083 (481-3005)	0.25	1000 (297-3594)	1371 (597-3034)	0.01
CK-MB (U/L)	112 (52-211)	120 (60-309)	0.33	138 (52-256)	174 (74-310)	< 0.01
Hs-Troponin T (ng/L)	2175 (592-5337)	2076 (912-5512)	0.70			
Troponin T (μg/L)				2.53 (0.55-7.63)	3.93 (1.39-8.67)	0.01

## Conclusions

Chronic metformin use in patients presenting with STEMI was associated with smaller infarct-size and not with lower MACE and mortality rates, as compared to other patients with diabetes.
